# Influence of the 2015–2016 El Niño on the record-breaking mangrove dieback along northern Australia coast

**DOI:** 10.1038/s41598-021-99313-w

**Published:** 2021-10-14

**Authors:** S. Abhik, Pandora Hope, Harry H. Hendon, Lindsay B. Hutley, Stephanie Johnson, Wasyl Drosdowsky, Josephine R. Brown, Norman C. Duke

**Affiliations:** 1 Bureau of Meteorology, Melbourne, Australia; 2 School of Earth, Atmosphere and Environment, Monash University, Clayton, Australia; 3 Research Institute for the Environment and Livelihoods, Charles Darwin University, Darwin, Australia; 4 Department of Ecology, Environment and Evolution, La Trobe University, Bundoora, Australia; 5 School of Geography, Earth and Atmospheric Sciences, University of Melbourne, Melbourne, Australia; 6 ARC Centre of Excellence for Climate Extremes, University of Melbourne, Melbourne, Australia; 7 Centre for Tropical Water and Aquatic Ecosystem Research, James Cook University, Townsville, Australia

**Keywords:** Natural hazards, Climate sciences, Atmospheric science, Ocean sciences, Environmental sciences, Environmental impact

## Abstract

This study investigates the underlying climate processes behind the largest recorded mangrove dieback event along the Gulf of Carpentaria coast in northern Australia in late 2015. Using satellite-derived fractional canopy cover (FCC), variation of the mangrove canopies during recent decades are studied, including a severe dieback during 2015–2016. The relationship between mangrove FCC and climate conditions is examined with a focus on the possible role of the 2015–2016 El Niño in altering favorable conditions sustaining the mangroves. The mangrove FCC is shown to be coherent with the low-frequency component of sea level height (SLH) variation related to the El Niño Southern Oscillation (ENSO) cycle in the equatorial Pacific. The SLH drop associated with the 2015–2016 El Niño is identified to be the crucial factor leading to the dieback event. A stronger SLH drop occurred during austral autumn and winter, when the SLH anomalies were about 12% stronger than the previous very strong El Niño events. The persistent SLH drop occurred in the dry season of the year when SLH was seasonally at its lowest, so potentially exposed the mangroves to unprecedented hostile conditions. The influence of other key climate factors is also discussed, and a multiple linear regression model is developed to understand the combined role of the important climate variables on the mangrove FCC variation.

## Introduction

**Figure 1:**
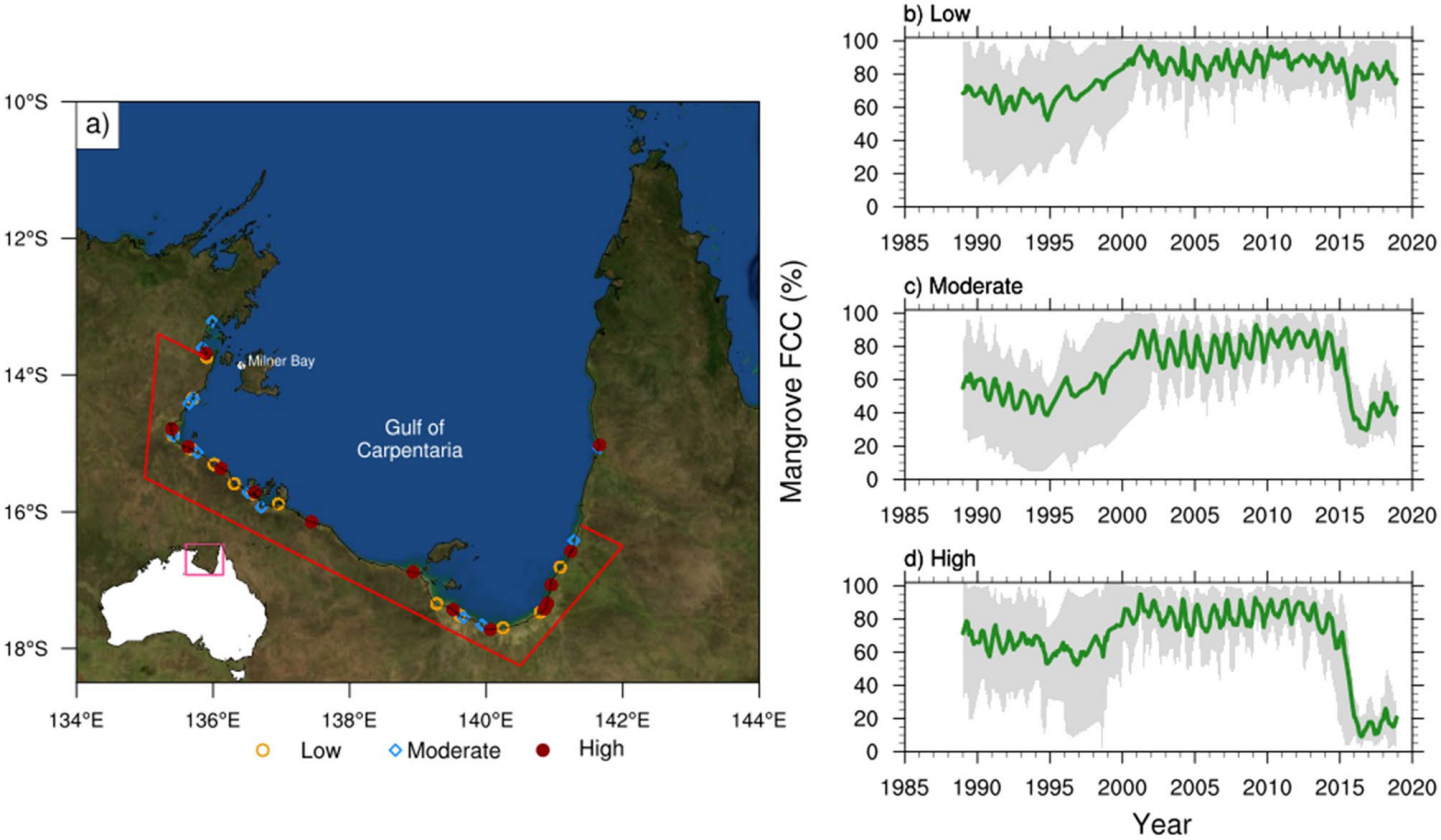
(**a**) Location of the mangrove FCC sampling sites along the Gulf of Carpentaria coastline with an inset in the bottom left showing the location of the Gulf of Carpentaria within Australia. The sites with three dieback classes—low (30–59%), moderate (60–79%), and high (80–100%) are marked with yellow circles, blue diamonds and maroon dots, respectively. The coastal Gulf region that is used to define $\hbox {T}_{{max}}$, rainfall and ESI anomalies are shown in red polygon. The basemap image is obtained from NASA Earth Observatory (https://visibleearth.nasa.gov/images/73909/december-blue-marble-next-generation-w-topography-and-bathymetry) and processed using the NCAR Command Language version 6.6.2 (http://www.ncl.ucar.edu/). (**b–d**) Time evolution of mangrove FCC (in %) for the three dieback categories. The solid green curves indicate the averaged FCC across all the sites in each category, while the range of the monthly FCC is shown with grey shading.

In late 2015, about 8000 hectares of the forested tidal wetland along a 1000 km stretch of the coastline of Australia’s Gulf of Carpentaria (see Fig. 1a for its location) experienced an extensive dieback of its shoreline mangroves^1–3^. Mangroves are an important part of the local ecosystem, providing habitat for many marine and coastal species, protecting coastlines from extreme weather and erosion, filtering out sediment from river run-off to protect seagrass, as well as sequestering significant levels of carbon in their sediments^4–6^. Because of their ecological importance, considerable attention has been paid to this dieback event to understand its extent, cause, and severity^7–9^. Mangrove dieback events were also observed during 2015–2016 in Kakadu National Park, Northern Territory^10^ and in a semi-arid stand near Exmouth, Western Australia^11^, but those events were not as extensive and severe as the Gulf of Carpentaria mangrove dieback. Preliminary observations have ruled out any direct influence of human activities (e.g., oil spills), pathogens, or any extreme weather event (e.g., tropical cyclone) at this time. Thus, the prevailing climate conditions, driven by the mega-El Niño that occurred during 2015–2016, might be the primary factor for the dieback^1,12^, but the precise connection with the climate drivers has not previously been identified.

Mangroves exhibit wide physiological tolerance and can utilize rainfall and groundwater as well as saline water during tidal inundation^13,14^, thus they can withstand a wide range of daily and seasonal hydrological variations. However, a major departure from the usual hydrological regime can induce physiological stress to the mangroves and their spatial extent may be considerably modified^11,15^. Earlier studies have suggested a range of climatic factors that might be important in causing stress to the mangroves, including unusually high temperatures, below-normal rainfall and humidity, and sustained below-normal sea level^1,12^. Harris et al.^16^ described the 2015 dieback event as a climatic ‘press-pulse’ process, with a long-term, slow climatic press changing the climatic envelope that ecosystems are adapted to, while the impact of the press amplified the extreme of pulse events. Overlaid on an ongoing warming trend associated with global warming, it is suggested that the ‘pulse’ was not sudden for the Gulf mangrove dieback but comprised a sustained months-long drop in sea level due to the 2015–2016 El Niño^12^, and enhanced air temperatures with persisting drought conditions acting to limit freshwater input to the coastline. Owing to their shallow root system ($< 0.5\hbox {m}$)^17^, common mangrove species, particularly *Avicennia marina* and to a lesser extent *Rhizophora stylosa*, are vulnerable to prolonged below-normal sea levels. Sippo et al.^18^ concluded that dry conditions combined with unprecedented below-normal sea levels and associated porewaters enriched in iron were the likely causes of the dieback. As a result of any prolonged lowering of sea level height (SLH), a change in the ecosystem can be triggered with a shift from mangrove forest to drier and more saline saltmarsh, where only a few specialized plants can survive^19^.

The Gulf of Carpentaria has a monsoonal climate with about 6 months of the dry season that extends from mid-April to mid-October^20^. If the lowering of SLH occurs during the late dry season following persistent below-normal wet season, a significant physiological water stress is likely to the mangroves^21^. However, mangroves are reasonably heat tolerant^22^ and the Gulf mangroves cope with the dry season every year. Furthermore, they have apparently remained mostly unaffected during other El Niño events in the recent past, even during previous strong El Niños such as the 1997–1998 event^1^. This evokes the questions: what was unusual about the 2015–2016 El Niño that resulted in the massive dieback? Was it simply the El Niño’s magnitude, or was the timing and possibly its co-occurrence with other climatic factors crucial? Any effort to address these outstanding questions will help improve our understanding of how such an environmental catastrophe occurred and should facilitate improved capability for monitoring and prediction. This would have a great value in the optimization of risk management for policymakers, rangers, and communities in the future.

In this study, we examine the key climate variables during the 2015–2016 El Niño in the context of conditions during previous El Niño events. We utilize satellite-based mangrove fractional green cover observations to investigate the variation of mangrove canopies associated with changes in SLH, rainfall, surface air temperature, and a measure of vegetation moisture stress formed by comparing potential and actual evapotranspiration. Based on the observed mangrove canopy change during previous El Niño events and any associated droughts and heat extremes, we provide a plausible explanation for the 2015 dieback that points to the seasonal timing and magnitude of the 2015–2016 mega El Niño as the primary driver. We also derive a statistical relationship between mangrove canopy changes and SLH, temperature, and evapotranspiration variations to provide a stress index model which can be used to flag future threatening climate conditions that may lead to mangrove dieback. We believe this to be an essential component of any coastal monitoring system, especially for similar tropical conditions.

## Data and method

The Gulf of Carpentaria is a large, shallow inlet from the Arafura Sea with limited coastal development and industry. Its shoreline consists of coastal plains of very low topographic relief with extensive saltpans, saltmarsh, and a mix of fringing, riverine, estuarine, and basin mangroves that grow along the seaward margin of the intertidal zone^17^. The mangrove stands dominated by *Avicennia marina* with seaward patches of *Rhizophora stylosa* trees and under-canopy occurrence of the shrub species *Aegialitis annulata*. The 2015–2016 dieback was most severe in the fringing mangrove communities with *A. marina* particularly impacted, while riverine and estuarine stands experienced the lowest levels of dieback^3^. The satellite-based Landsat Thematic Mapper (TM), enhanced TM and operational land imager provide 25 m spatial resolution observation of mangrove fractional green cover every 16 days (cloud permitting) since the late 1980s^23^. The vegetation cover is estimated at sampling sites that are randomly located within the maximum historical extent of the mangroves (Fig. 1a). To establish pre-dieback limits of mangrove community extent, the cumulative mangrove area has been mapped across five years prior to the 2015–2016 dieback event using annual cover sequence products from the Mangrove canopy cover v2.0.2^24^. This extent is used to (i) constrain a randomly generated sampling dataset and (ii) ensure that sampling does not capture any non-target vegetation species. The random points dataset is used to position 42 sampling sites (Fig. 1a) distributed across the dieback regions of the Gulf as defined by Duke et al^1^. The 90 $\times$ 90 m sites are designed to cover multiple pixels and account for spatial variability with missing data due to the high likelihood of seasonal cloud cover. For each site, fractional canopy cover (FCC) time-series are compiled using all available scenes from Digital Earth Australia’s (DEA) fractional cover archives ($\sim$ 25 m). FCC estimates are retrieved from Landsat pixels from Landsat 5 (TM), 7 (ETM+), and 8 (OLI), which are decomposed into estimated fractions of photosynthetic vegetation, non-photosynthetic vegetation, and bare ground, using spectral unmixing algorithms developed by the Joint Remote Sensing Research Program. FCC scenes are clipped to the site bounds and bitwise flags of the DEA’s Water Observations from Space product are used to mask pixels affected by cloud, cloud shadow, and open water before extracting fractional cover values for each site. This process is similar to cloud-filtering routinely implemented for Landsat datasets^25^. Time-series responses are visually inspected for relative FCC change and categorized as high (80–100%), moderate (60–79%), and low (30–59%) severity based on the relative FCC change calculated between pre-dieback (preceding dry season of 2015) and post-dieback (following wet season of 2015–2016). Outliers are excluded from the time-series using a rolling mean to detect data points that exceeded more than 20% FCC change from the three-month average. All fractional cover products are extracted using DEA’s sandbox platform using notebook and tool repositories developed by the DEA, and the products are accessed from the open data^26^.

The diurnal tidal range in the Gulf of Carpentaria is largely classified as meso-tidal ($\sim 2$–3 m)^27^. As the daily mean tidal residual of the SLH are similar across the stations in Gulf^28^, it is reasonable to use a single tide-gauge location as representative of SLH in the Gulf of Carpentaria. Monthly SLH variations are monitored with observations from the Australian Bureau of Meteorology (BOM) Milner Bay, Groote Eylandt tide-gauge station (location shown in Fig. 1a) during 1993–2018. In order to understand the spatial SLH variation, we also utilize monthly Bluelink ReANalysis (BRAN) SLH analyses^29^, which are available globally between ${75}^{\circ }S-{75}^{\circ }N$ at $\sim$10km resolution. BRAN assimilates observations of SLH (based on satellite altimetry and in situ observations) together with in situ observations of temperature and salinity into a global ocean model using ensemble optimal interpolation of observational data assimilation system to provide several 3-dimensional time-varying analyses of ocean temperature, salinity, currents, and SLH.

Monthly rainfall and maximum surface air temperature ($\hbox {T}_{{max}}$) dataset with a horizontal resolution of $5km \times 5km$ are obtained from the Australian Water Availability Project (AWAP)^30^. This data is available across continental Australia back to 1911. We use monthly surface zonal wind analyses from European Centre for Medium-Range Weather Forecasts (ECMWF) Interim reanalysis (ERA-I)^31^, which are available globally on a ${1.5}^{\circ }\times {1.5}^{\circ }$ grid during 1979–Aug 2019. An evaporative stress index (ESI)^32,33^ is used to examine the evaporative stress along the Gulf’s coast. This index is the normalized ratio of evapotranspiration to potential evapotranspiration, which are derived from the outputs of the AWRA-L landscape water balance model over Australia (version 6) and indicative of surface moisture supply and evaporative demand^34^. The rainfall, $\hbox {T}_{{max}}$ and ESI data are averaged over the coastal Gulf region (shown as a red box in Fig. 1a).

The anomalies are calculated by removing the seasonal cycle (time mean and first three harmonics of the climatological annual cycle) from the variable and the linear trends are removed from the anomalous time-series. The anomalies are standardized by their own standard deviation to obtain the normalized anomalies. We identify previous strong El Niño Southern Oscillation (ENSO) events as when the oceanic Niño3.4 index exceeds 1.5 standard deviations in the November–January season, when El Niño conditions typically peak in central to the eastern equatorial Pacific. This Niño3.4 index is an average of sea surface temperature (SST) over the central Pacific region (${5}^{\circ }$S–${5}^{\circ }N$, ${170}^{\circ }$W–${120}^{\circ }W$), calculated using HadISST dataset^35^. To increase the sample size of strong El Niño events, we assume linearity of La Niña events (being opposite to El Niño) and include in our composite of the previous strong La Niña events but flip the sign of the anomalies. Based on this index, we identify the austral summers of 1957–1958, 1965–1966, 1972–1973, 1982–1983, 1987–1988, 1991–1992, 1997–1998, 2015–2016 as strong El Niño years and 1973–1974, 1975–1976, 1988–1989, 1998–1999, 1999–2000, 2007–2008, 2010–2011 as strong La Niña years. The Interdecadal Pacific Oscillation (IPO) is monitored using SST-based IPO tripole index (TPI)^36^.

## Results

### Historical perspective of the mangrove FCC variability

The monthly time-series of the mangrove FCC in the three dieback categories for over 30 years are displayed in Fig. 1b–d. The FCC dataset is averaged over all the sites in each category and the mean monthly FCC time-series with its range across the sites are shown. A substantial drop in FCC is evident for the moderate and high dieback categories during 2015, with some locations in the high category experiencing near 100% loss of FCC. However, abundant variability is also evident, primarily associated with the seasonal cycle (FCC peaks in austral autumn), but also with other low-frequency variations. For instance, the FCC has a sustained 20% reduction around 1994 and subsequent recovery during the late 1990s. However, the magnitude of the drop during 2015 is unprecedented at least in the last few decades (e.g., Duke et al.^1^). The seasonal variation of the mangrove FCC is $\sim 10\%$ of the mean, with the maxima occurring in March–April and the minima around October (Figure S1). During 2015, the seasonal reduction of FCC started about a month or two before the typical seasonal minima and it dropped noticeably for moderate and high categories by August–September.

### Impact of El Niño events

**Figure 2:**
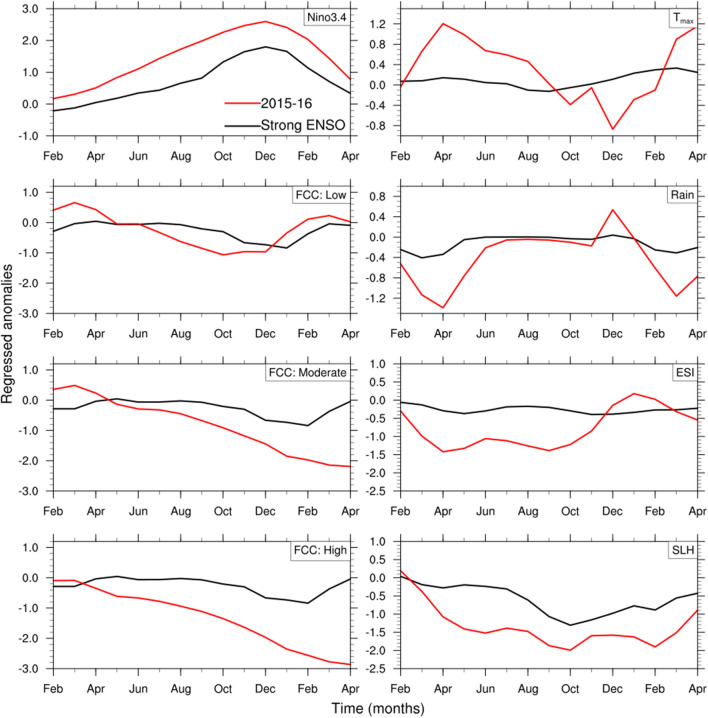
Monthly evolution of Niño3.4, mangrove FCC for 3 dieback categories, $\hbox {T}_{{max}}$, rainfall, ESI, and sea level height (SLH) anomalies during previous strong El Niño conditions (black curve) and 2015–2016 (red). All the variables are normalized by their own standard deviation before lag-regressing the normalized anomalies onto Niño3.4 and the regression coefficients are scaled by normalized Niño3.4 index magnitude during strong ENSO. Plots are generated using the NCAR Command Language version 6.6.2 (http://www.ncl.ucar.edu/).

Considering the coincidence of the strong El Niño of 2015–2016 with the 2015 dieback event, we examine the variation of different climate variables during the 2015–2016 El Niño and compare them with previous strong El Niño conditions. This provides a framework for understanding the extreme climate conditions that might have stressed the mangroves during 2015–2016. Figure 2 shows the evolution of the normalized Niño3.4 index, mangrove FCC, $\hbox {T}_{{max}}$, rainfall, ESI along the Gulf coast, and SLH anomalies at Milner Bay from the development period of the strong El Niño events (the preceding February) to their demise (the following April). The influence of strong El Niños on the variables is obtained by lag-regressing the normalized anomalies onto December Niño3.4 index and the derived regression coefficients are scaled by the normalized Niño3.4 index magnitude (1.44) during strong ENSO. It is noteworthy to mention that SST in the Niño3.4 region of the eastern equatorial Pacific tends to peak in December. Mangrove FCC reduces after the peak of El Niño events, but this reduction in FCC is not usually large. During a typical El Niño event, $\hbox {T}_{{max}}$ in the Gulf region is near to neutral in the development stage and then it becomes higher than normal at the peak of El Niño (i.e., during December–February). For this Gulf region, rainfall displays little variation during El Niño, however evaporative stress is typically stronger than normal (i.e., negative anomalies) throughout the course of El Niño. Finally, SLH in the Gulf is also seen to be typically lower than normal from late winter through to early autumn, consistent with the strong influence of the Pacific on the Gulf SLH variation as described in Oliver and Thompson^28^.

During the 2015–2016 El Niño, the Niño3.4 SST index was 50% stronger compared to the composite at its peak in December 2015. It started to become positive earlier since the preceding austral winter and lasted later into the following autumn. A strong positive $\hbox {T}_{{max}}$ anomaly developed around April–May, but it reversed the sign during the peak of the event. Lower than normal rainfall accompanied the high $\hbox {T}_{{max}}$ in the preceding winter, but rainfall was near normal during the peak of the event during spring and early summer 2015, consistent with the weak rainfall anomalies observed during previous strong El Niño events. And, in contrast to other El Niño events, ESI was very negative in the autumn, winter, and spring of 2015, indicating the persistence of a stressful environment for the mangroves. Lastly, SLH became much lower than normal 4–5 months earlier than is typically observed during El Niño and remained lower longer into the following autumn. The detrended SLH time-series at Milner Bay indicate that the drop during 2015 was unprecedented. From this cursory analysis, the extreme drop in SLH together with the preceding strong increase in negative ESI and high $\hbox {T}_{{max}}$ during the preceding winter could have provided the necessary stress to cause the extreme dieback that was sustained during 2015 (Fig. 2). The persistent below-normal SLH especially during hot and dry conditions would have resulted in limited tidal inundation resulting in reduced soil water contents^14,18^.

### Connections between mangrove FCC and climate variables

We further explore the historical relationship between SLH and mangrove FCC and their association with El Niño conditions in the equatorial Pacific using cross-spectral analysis between SLH and FCC. This analysis is useful for quantifying the relationship between two variables as a function of frequency and estimating their phase relationship. We compute the coherence squared by Fourier transforming the deseasonalized monthly time-series for 1994–2016, which provides a bandwidth of 1/276 $\hbox {month}^{-1}$. The coherence squared is formed after smoothing the raw power and cross power estimates with a 9-point box car, yielding an effective bandwidth of 9/276 $\hbox {month}^{-1}$.

**Figure 3:**
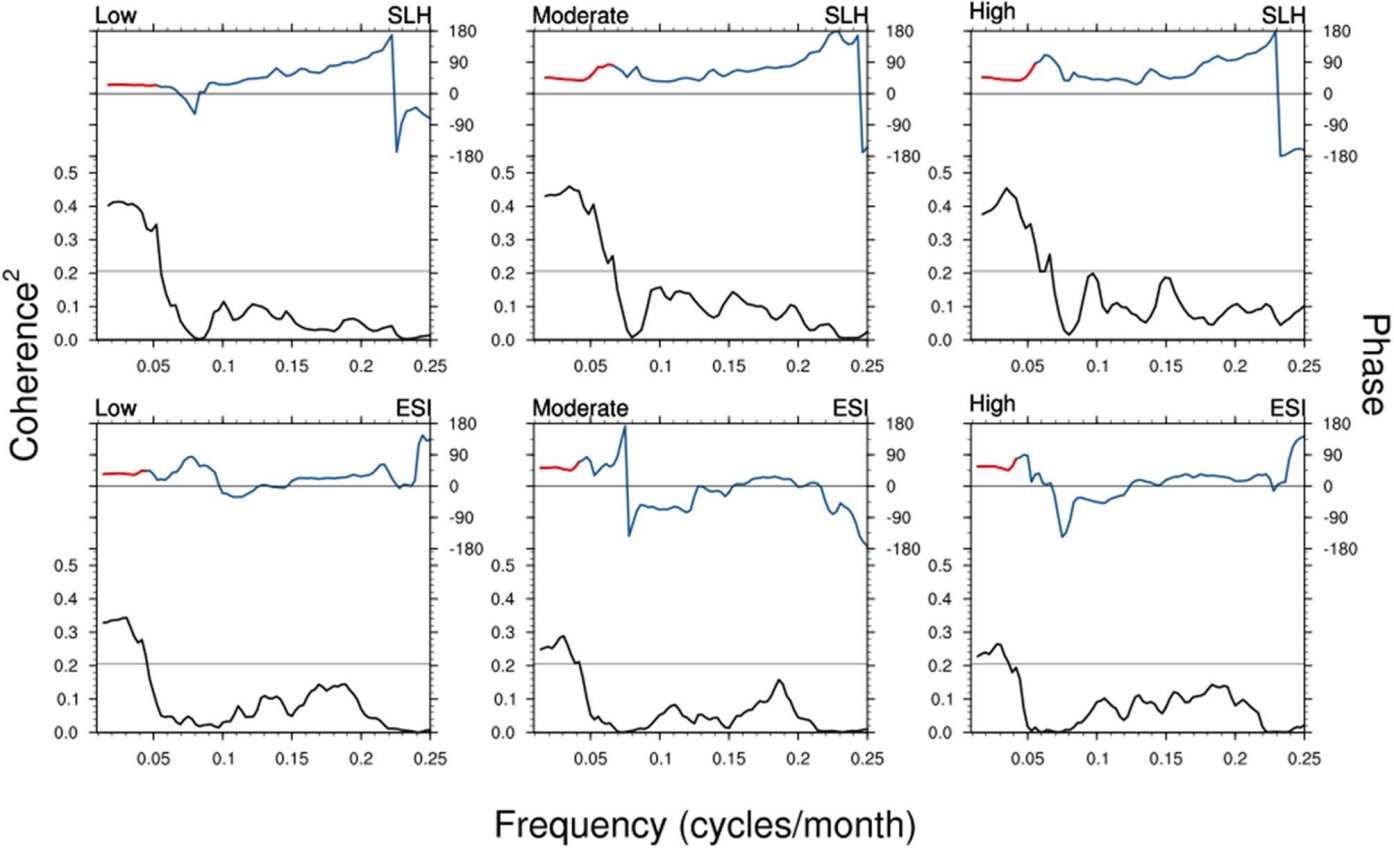
The coherence-squared spectrum (black curve, below) and phase (blue curve, top) between sea level height (SLH) at Milner Bay and mangrove FCC for the three dieback categories of low, moderate, and high (top panels). The same with coastal Gulf area-averaged ESI is shown at the bottom panels. The gray line shows a 5% level of significance for coherence-squared values. Phase lags are shown in red where the coherence squared is significant at 5% level. Plots are generated using the NCAR Command Language version 6.6.2 (http://www.ncl.ucar.edu/).

The top panels of Fig. 3 show the coherence squared and phase lag between the monthly SLH at Milner Bay and the index of mangrove FCC for three dieback categories. The cross-spectra are found to be largely similar for all three dieback categories - the peak coherence up to 0.45 occurs around 0.03 $\hbox {month}^{-1}$ frequency (period $\sim 2.8 \,\hbox {years}$) and are broadly high for frequencies lower than 1/25 $\hbox {month}^{-1}$ (period $\sim 2.1\, \hbox {years}$). There are no other significant spectral peaks at higher frequencies. The phase lag (about ${45}^{\circ }$) indicates that low frequency (period longer than 2 years) FCC variation lags SLH by about 5–6 months, which is reasonable as it should take a few months for the mangroves to react to lower or higher than normal sustained SLH change.

A similar quantification of the relationship between Gulf’s mangrove FCC and coastal Gulf region area-averaged ESI is shown at the bottom panels of Fig. 3. A weaker spectral peak at periods longer than 2 years is also evident with about 3–4 months’ phase lag. We also examine the coherence-squared between FCC and $\hbox {T}_{{max}}$, and rainfall (Figure S2) and unlike SLH and ESI, these variables generally show no significant coherence with FCC. Only the peak coherence between $\hbox {T}_{{max}}$ and FCC for moderate and high dieback categories at frequencies lower than 24 months is found to be marginally significant ($\hbox {p}=10\%$). An out-of-phase relationship is also noted between FCC and $\hbox {T}_{{max}}$, indicating a reduction of FCC due to low-frequency warming over the Gulf.

**Figure 4:**
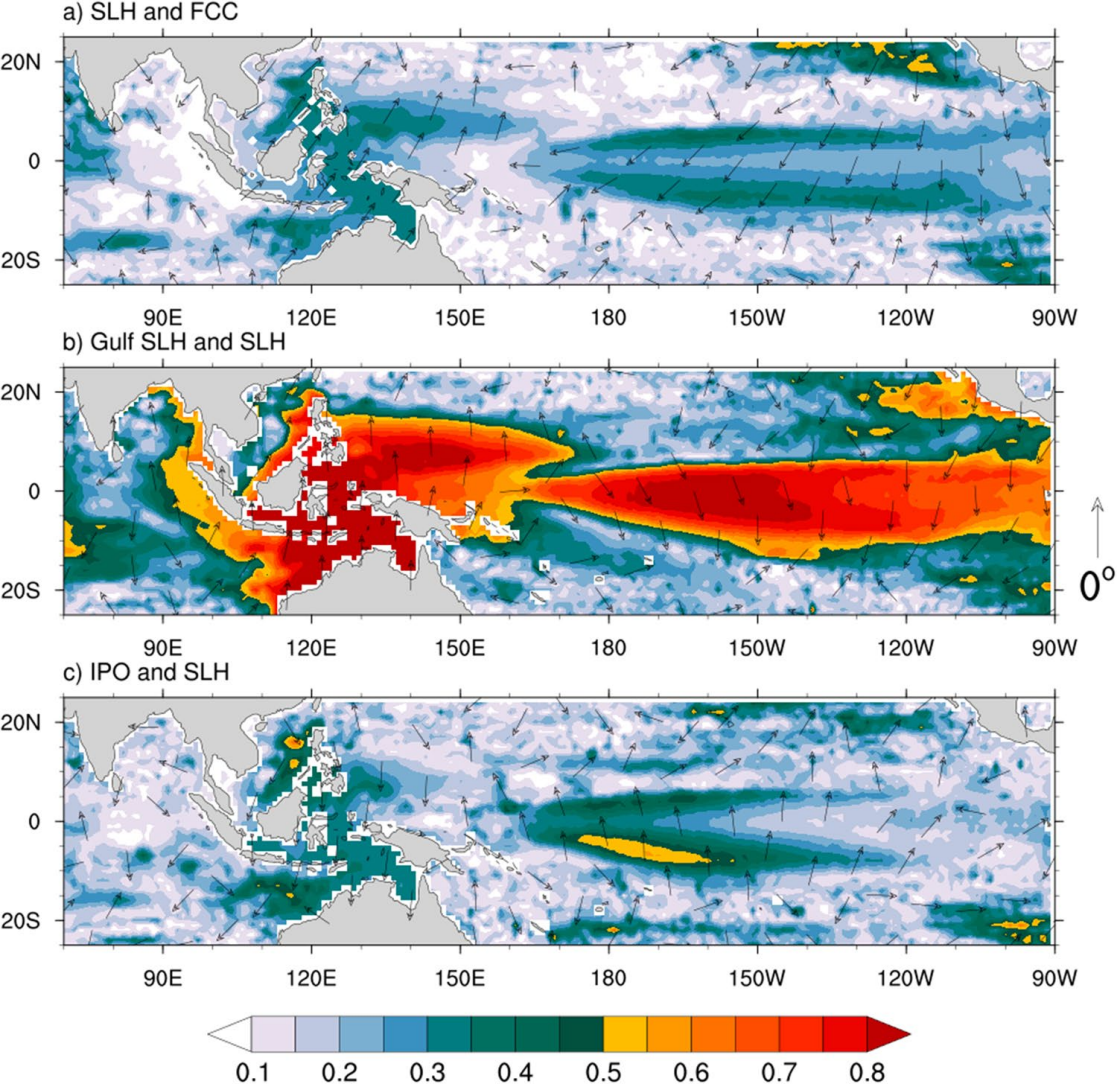
Spatial distribution of the squared coherence at the dominant frequency mode of cross-spectra (shaded) and phase difference (vectors) between (**a**) sea level height (SLH) and averaged mangrove FCC for three dieback categories. (**b**) Gulf (Milner Bay) SLH and gridded SLH dataset, (**c**) IPO and SLH. Vectors pointing north represent no phase lag and the lag increases in the clockwise direction. The positive lag implies mangrove FCC lags SLH anomalies and vice versa. Plots are generated using the NCAR Command Language version 6.6.2 (http://www.ncl.ucar.edu/).

More insight into the cause of the low-frequency SLH anomalies associated with FCC variations is gained by examining the spatial distribution of the coherence-squared between the gridded BRAN SLH and mangrove FCC index anomalies. The coherence-squared and phase lag for the dominant low-frequency band around the 30-month cycle are shown in Fig. 4a. Here we form the index of FCC by averaging the standardized FCC anomalies for the three dieback categories. Mangrove FCC at Gulf of Carpentaria sites is coherent with SLH throughout the Gulf and on the north-west coast of Australia, in the seas of the Indonesian archipelago, and in the north-west Pacific, with a constant phase lag of about 1/8 cycle ($\sim$4 months). The FCC is also found to be coherent with SLH on the equatorial central and eastern Pacific, with a distinctive horseshoe pattern. The phase is roughly ${180}^{\circ }$ shifted from that in the Gulf and surrounding seas, indicating that FCC is coherent with an El Niño-like fluctuation of SLH (i.e., low SLH in the Gulf and western Pacific coincides with high SLH in the central and eastern Pacific). However, the pattern of coherence in the central and eastern Pacific, with its distinctive horseshoe shape, is more indicative of the interdecadal variation of ENSO referred to as the IPO (e.g., Power et al.^37^). This sensitivity of FCC to the lower frequency component of ENSO presumably arises for two reasons. First, the lower frequency component of ENSO drives a stronger variation of SLH in the Gulf than it does by the typical interannually varying El Niño event. This is confirmed by examining the coherence of SLH in the Gulf with SLH at every grid point for the dominant frequency band (Fig. 4b). The peak coherence with SLH in the eastern Pacific, which has the opposite phase to that in the Gulf, occurs just east of the dateline and with a relative minimum in coherence along the equator in the eastern Pacific. This is a typical IPO feature and the coherence between the IPO index and SLH confirms this structure (Fig. 4c). Secondly, as FCC is effectively a slowly varying parameter, it is more coherent with the lower frequency components of El Niño. These two processes together result in a pattern of coherence between FCC and SLH that looks more like the IPO than it does El Niño. That is, FCC is more responsive to the low-frequency tail of ENSO variability than it is to the higher frequency interannual components.

The overall pattern of coherence of SLH with FCC thus reflects the spatial structure of ENSO, suggesting that the El Niño driven SLH variability does influence mangrove canopy evolution. During El Niño, the westerly wind anomalies in the western Pacific act to elevate the SLH to the east with a Kelvin wave structure and lower SLH in the western Pacific with Rossby wave structure^38^. The lower SLH anomalies in the west Pacific travel through the Indonesian seas and eventually act to lower the SLH in the Gulf^28^ and down the west coast of Australia where it is transmitted as a coastally trapped Kelvin wave^39^. The broader meridional structure of the SLH variation in the central and eastern Pacific is coherent with Gulf FCC and that reflects the structure of the lower frequency tail of El Niño, and it is associated with slower westward propagating Rossby waves off the equator in the eastern and central Pacific. However, it remains in question why the mangroves dramatically declined in 2015 but survived during other El Niño events. We explore this outstanding question by examining SLH variation during strong El Niño events next.

### Sea level variation during strong El Niños—how did 2015–2016 differ?

**Figure 5:**
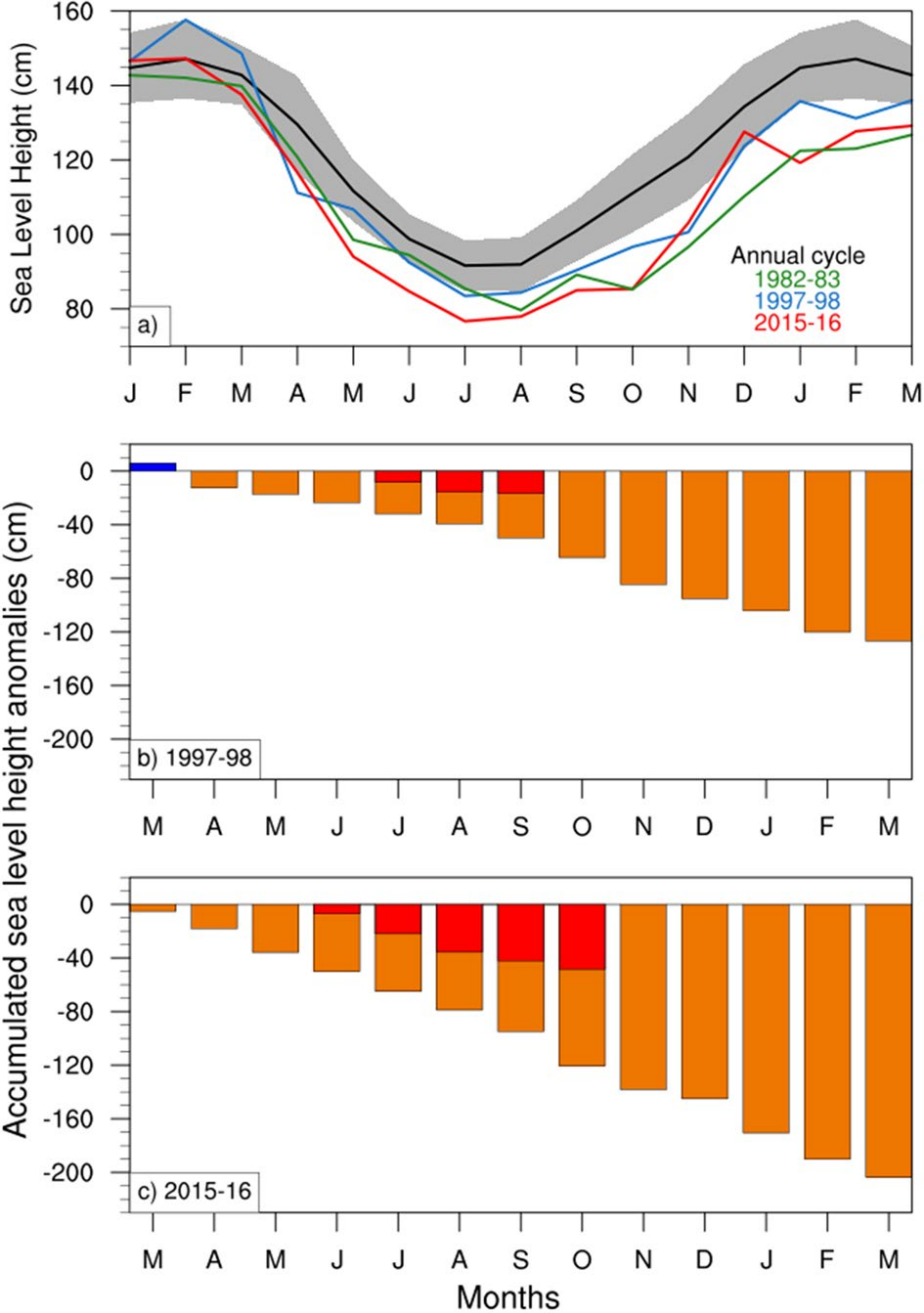
(**a**) Sea level height (SLH, in cm) evolution at the Gulf of Carpentaria during Strong El Niño events, along with the climatological annual cycle (black curve) and its two-standard deviation (2$\sigma$) range (grey shading). (**b,c**) Accumulation of drop-in SLH anomalies during two recent strong El Niño events (1997-1998 and 2015-2016, respectively) are shown as orange bars. The similar accumulation relative to climatological minima is denoted by red bars. The linear trend is removed from the SLH data before the analysis. Plots are generated using the NCAR Command Language version 6.6.2 (http://www.ncl.ucar.edu/).

To gain insight into the SLH variation during El Niños, we look at the timing of the SLH decline during 2015–2016 and compare that with other strong El Niño events. Figure 5a displays the annual cycle of SLH in Milner Bay along with the variations during 2015–2016 and two other strong El Niño events (1982–1983 and 1997–1998). The SLH maximum occurs during austral summer and the minimum is noted during late winter. Forbes and Church^40^ explained seasonal SLH variation as a result of the seasonal cycle of wind stress over the region (i.e., westerlies during the summer monsoon acting to raise SLH and peak easterly trade winds during winter that act to lower SLH). The three El Niño events shown in Fig. 5a result in lower-than-normal SLH from about April of the year El Niño commences through to the following March. But it is noted that no severe mangrove dieback was recorded during the 1997–1998 El Niño event, which has been referred to as “El Niño of the century” for its extraordinary magnitude and influence on the global weather and climate^41^. The most dramatically different behavior of the 2015–2016 El Niño is the much stronger negative SLH anomalies during austral autumn and winter, when the anomalies were more than 12% stronger than the previous two very strong El Niño events. The extreme sea level decline in 2015 coincided with the seasonal minimum, so potentially exposed the mangroves to unprecedented low SLH.

The extremity of the low SLH during 2015 is quantified by computing the cumulative deficit of detrended SLH. It is done for both cumulative anomaly and cumulative anomaly below the climatological minimum (assumed to be the threshold below which the mangroves cannot be sustained). We show this for the 1997–1998 (Fig. 5b) and 2015–2016 (Fig. 5c) El Niño events. Our analysis indicates that cumulative stress due to below-normal SLH was double in 2015–2016 compared to 1997–1998. Sustained SLH below the climatological minimum continued into October 2015 but was confined to only moderate values during June–August 1997.

**Figure 6:**
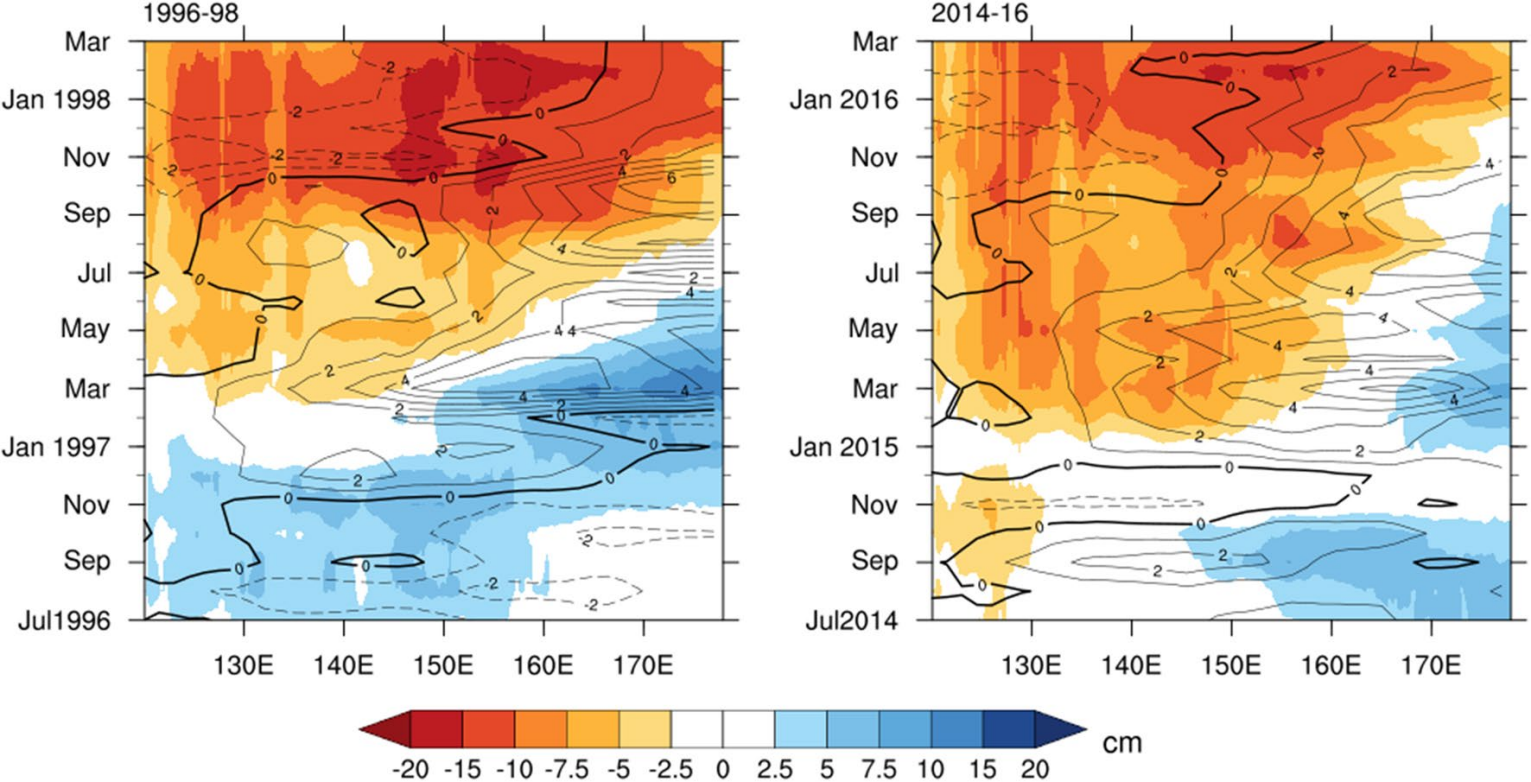
Time-longitude plot of BRAN sea level height (shaded, cm) and ERA-interim surface zonal wind (contours, m $\hbox {s}^{-1}$) anomalies averaged between ${5}^{\circ }$S and ${5}^{\circ }$N for two El Niño events during 1996–1998 and 2014–2016, respectively. Plots are generated using the NCAR Command Language version 6.6.2 (http://www.ncl.ucar.edu/).

We further evaluate the SLH and surface zonal wind evolution around the equatorial Pacific during the strong El Niño events of 1997–1998 and 2015–2016 (Fig. 6). This domain was chosen as the onset of the wind anomalies occur along the equator^42^ and the wind-forced SLH changes in the equatorial western Pacific appear to have a strong relationship with the Gulf’s SLH variation. Figure 6 indicates that the drop in SLH around the equatorial Pacific during peak El Niño months of 1997–1998 was stronger relative to the same period of 2015–2016. But during the early and middle months of 2015, the SLH drop was greater compared to the same period in 1997. The below-normal SLH anomalies during 2015 can be traced back to mid-2014, when an El Niño event false-started^43^. As the westerly zonal wind anomalies strengthened around late austral autumn of 2015, the wind-induced downwelling Kelvin wave acted to lower SLH in the western Pacific for a prolonged period in the early and middle months of 2015, especially in the dry season of the year.

Our analysis suggests that the low-frequency (periods longer than 2–3 years) SLH was the key driver of the mangrove dieback event. A persistent drop in SLH associated with El Niño likely established a stressful environment for the mangroves, especially during the dry months of the year (late austral autumn and winter). Additionally, the evaporative stress, as quantified by the ESI, appears to be vital for the mangroves as does $\hbox {T}_{{max}}$. The 2015 El Niño was thus catastrophic because SLH dropped much earlier than that usually occurs during El Niño. It exposed the mangroves to a sustained period of SLH well below its climatological minimum, together with atmospheric anomalies that resulted in increased evaporative stress during the preceding autumn and winter seasons. This sustained low relative sea level was captured by the mangrove stress index defined in Duke et al.^3^. In this study, we go further and include other potential factors to derive a mangrove stress index.

### Mangrove stress index

To understand the combined role of the potential climate variables on the dieback event, we reconstruct the mangrove FCC anomalies for each of the dieback categories using SLH, ESI and $\hbox {T}_{{max}}$ anomalies. A multiple linear regression model is developed to estimate the FCC variations with seasonal cycle removed, and detrended, standardized 6-month lagged SLH at Milner Bay, 3-month lagged ESI, and $\hbox {T}_{{max}}$ anomaly time-series over the coastal region of the Gulf as the predictors. A 12-monthly running mean is applied to the predictors to eliminate the high-frequency variability. The mathematical model for the multilinear regression analysis is as follows:

1 $$\begin{aligned} FCC_{estimated} = b_{0} + b_{1}.SLH + b_{2}.ESI + b_{3}.T_{max} \end{aligned}$$

**Figure 7:**
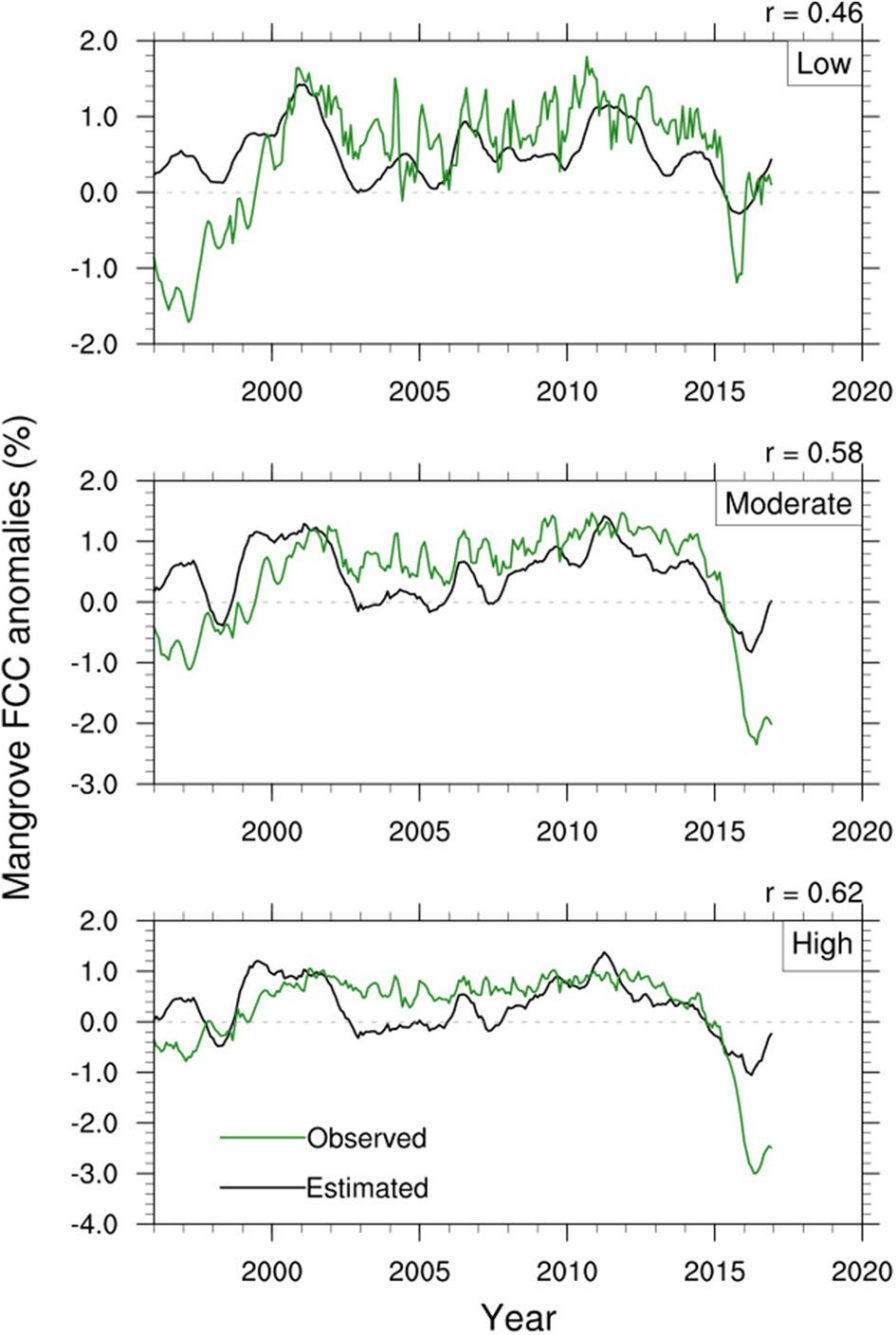
Reconstruction of mangrove FCC anomalies (seasonal cycle removed) using multilinear regression of 6-month lagged SLH, 3-month lagged ESI and $\hbox {T}_{{max}}$ anomalies (seasonal cycle removed) averaged over Gulf of Carpentaria coastal region. The predictors are detrended and a 12-monthly running mean is applied to filter out the high-frequency noise. All the datasets are normalized by their own standard deviation. Note that Y-axis ranges are not the same in all panels. Plots are generated using the NCAR Command Language version 6.6.2 (http://www.ncl.ucar.edu/).

The estimated and observed FCC for three dieback categories over the period of 1994–2018 are displayed in Fig. 7 and the associated regression coefficients with relevant statistics are given in Table 1. Consistent with our previous results, SLH has the dominant contribution, and the associated coefficients are significant at the 5% level for all three dieback categories. By contrast, temperature and ESI show weaker association and the regression coefficients for the $\hbox {T}_{{max}}$ anomalies are not significant at the same confidence level.

The correlations between estimated and observed FCC range between 0.47 and 0.62. Relatively higher correlations are noted for the high dieback, suggesting adequate skill of the regression model in representing the vulnerable mangrove FCC. The reconstructed FCC also shows an unprecedented reduction in FCC during 2015, though the estimated decrease is much weaker than the observed. Nevertheless, the analysis in Fig. 7 confirms that the multilinear regression model can be used to construct the mangrove stress index for estimating the future condition of the mangroves using dynamical seasonal forecasts (e.g., Long et al.^44^) and climate model projections, and it will form the focus of future work.

**Table 1:** Regression coefficients for three dieback categories during 1994–2018 from the multiple linear regression model (1). Regression coefficients significant at 5% level are marked in bold.

Dieback class	Regression coefficients			
	$\hbox {b}_0$	$\hbox {b}_1$	$\hbox {b}_2$	$\hbox {b}_3$
Low	0.151	**0.427**	**0.386**	$-$ 0.076
Moderate	**0.377**	**0.606**	**0.12**	$-$ 0.089
High	**0.216**	**0.604**	**0.325**	$-$ 0.087

## Discussion

We investigate the key climate conditions during the record-breaking 2015 mangrove dieback event along the southern coastline of the Gulf of Carpentaria using available observations and reanalysis datasets. The decline in SLH associated with the positive phase of ENSO is shown to be primarily responsible for the dieback event. The eastward shift of the equatorial Walker circulation during the 2015–2016 El Niño drove a decline in sea level throughout the Gulf of Carpentaria, which potentially exposed the mangroves to hostile conditions. Our analysis over the period 1994–2018 suggests that low-frequency SLH variability (i.e. a period longer than 2 years) associated with ENSO explains about 50% variation of the observed low-frequency mangrove FCC variation. Slowly varying FCC is shown to be more strongly associated with the lower frequency (decadal) component of ENSO than its higher frequency interannual components.

The phase lag between SLH change and the variation of mangrove FCC (FCC declines after the SLH drop occurs) indicates that FCC reduces about 5–6 months after the persistent drop in SLH. During 2015, a much stronger SLH drop, about 12% stronger than previous strong El Niño events, and much earlier onset of the SLH decline (SLH dropped in June–July 2015 whereas typical SLH drop during El Niño does not occur until Aug–Sep) occurred during June–July 2015 when SLH was seasonally at its lowest, and that potentially exposed the mangroves to unprecedented lower water availability conditions. A prolonged period of low SLH coupled with stronger dry and warmer conditions during the 2015–2016 El Niño resulted in a highly stressful environment, caused the mass dieback of the mangroves.

The drop in SLH in the Gulf in 2015 was also associated with mangrove FCC reduction and dieback across the wider northern coastline of Australia^11,45^. This synchronous wider influence reflects the rapid adjustment of SLH along the north and west coasts of Australia in response to El Niño (e.g., Potemra^46^). The below normal SLH that began in 2014 due to weak El Niño conditions in that year were further lowered in the early and middle months of 2015 by the strong westerly wind anomalies around the equatorial western Pacific leading to critically below-normal SLH in the Gulf of Carpentaria during the dry season of 2015. The mechanism behind this remote response of sea level during El Niño in the Gulf (e.g., Oliver and Thompson^28^), along the Western Australia coast is well established and what set the 2015 El Niño apart from previous events. This event was distinguished by its strong amplitude and its early onset.

The severity of the 2015 mangrove dieback in the Gulf of Carpentaria is found to differ with geographical location, primarily due to distance to the shoreline, local elevation, and distance from catchment drainage channels^2,3^. There was a detectable impact of the 1997–1998 El Niño on the FCC at a few sites, causing $\sim$ 20–40% loss of FCC of the mangroves. In contrast, some of the sites were almost unaffected over the last few decades including during the 2015–2016 dieback event. A couple of sites in frontal stands, dominated by *Rhizophora stylosa*, sustained extensive dieback in 2015, while other unaffected sites occurred in relatively broad and lush areas next to tidal creeks. The remaining dieback sites were at least a few meters (60–200 m) away from the shoreline and had minimal elevation above the mean SLH. These contrasting patterns of dieback at fine-scales emphasize the importance of the individual hydro-geomorphic settings (defined by height above mean sea level, distance to drainage channels) and hydroperiod (duration of tidal inundation) as important factors in determining the severity of mangrove dieback. Further assessment of the site-scale geomorphic variation is beyond the scope of this present study.

It may be inferred from our analyses and preliminary assessment of Duke et al.^1^ that the 2015 mangrove dieback event was primarily driven by large-scale stressors (e.g., decadal ENSO variability), however, regional variation of mangrove species, inundation period, distance to drainage channels influenced extent of the dieback. This dieback event has a number of similarities with the 2000 saltmarsh dieback on the coast of Louisiana, USA in that multiple drivers (low mean sea level, below-average rainfall, and low river discharge) resulted in severe dieback of saltmarsh vegetation with severity determined by micro-topographical differences^47^. With the global warming^48^, and the changing features of ENSO^49^, mangroves are likely to experience further stress in coming decades^18^. The future investigation could include an examination of changes in the large-scale climate stressors in climate model projections to determine if there is an increased likelihood of experiencing mangrove stress events similar to 2015 in the Gulf of Carpentaria in a warming climate.

## Supplementary Information

Supplementary Figures.
