# Supplementary material for: Influence of the 2015–2016 El Niño on the record-breaking mangrove dieback along northern Australia coast
Source: Sci Rep. 2021 Oct 14;11:20411. doi: 10.1038/s41598-021-99313-w (PMC8516887; doi:10.1038/s41598-021-99313-w)
Supplement: Supplementary file 1 — Supplementary Figures. [file 41598_2021_99313_MOESM1_ESM.pdf]

Supplementary Information

Influence of the 2015-16 El Niño on the record-breaking mangrove dieback along northern Australia coast

S. Abhik[1], Pandora Hope[1], Harry H. Hendon[1,2], Lindsay B. Hutley[3], Stephanie Johnson[4], Wasyl Drosdowsky[1], Josephine R. Brown[5,6], & Norman C. Duke[7]

[1] Bureau of Meteorology, Melbourne, Australia
[2]School of Earth, Atmosphere & Environment, Monash University, Clayton, Victoria, Australia
[3] Research Institute for the Environment and Livelihoods, Charles Darwin University, Darwin, Australia.
[4]Department of Ecology, Environment and Evolution, La Trobe University, Bundoora, Victoria, Australia.
[5]School of Geography, Earth and Atmospheric Sciences, University of Melbourne, Melbourne, Australia.
[6]ARC Centre of Excellence for Climate Extremes, University of Melbourne, Melbourne, Australia
[7]James Cook University, TropWATER – Centre for Tropical Water and Aquatic Ecosystem Research, Townsville, Australia

E-mail: `abhik.climate@gmail.com`

Content: Fig.S1, Fig.S2

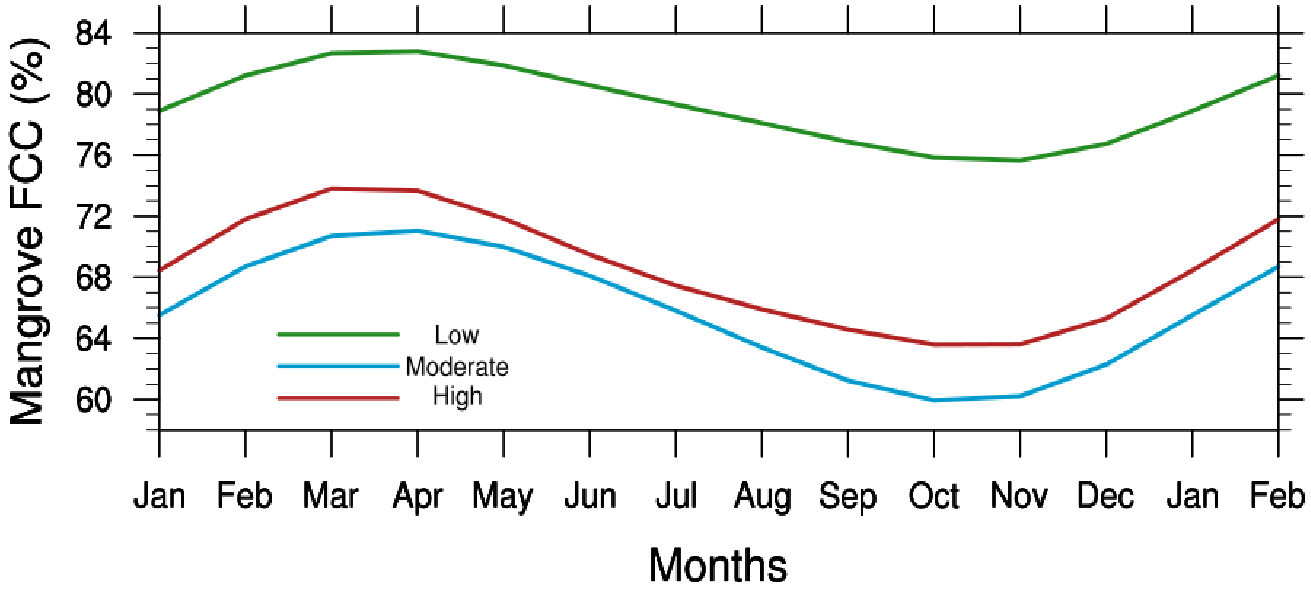

Figure S1. The seasonal cycle of mangrove fractional canopy cover (FCC) for the three dieback categories: Low, Moderate, and High (based on data from 1988-2019).

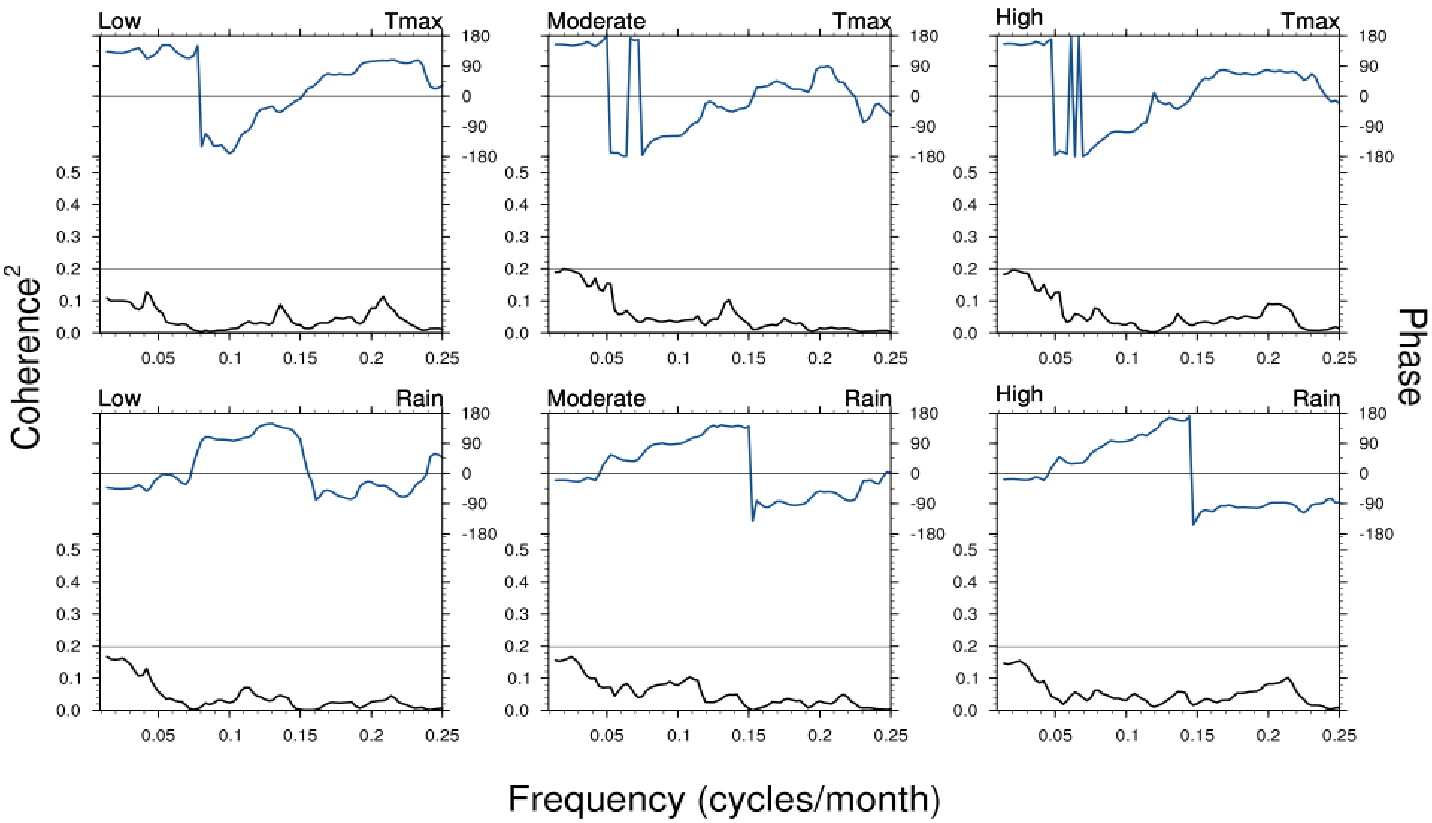

Figure S2. The coherence-squared spectrum (black curve, below) and phase (blue curve, top) between coastal Gulf area-averaged T_{max} and mangrove FCC for the three dieback categories of low, moderate, and high (top panels). The same with coastal Gulf area-averaged rainfall is shown at the bottom panels. The gray line shows a 5% level of significance for coherence-squared values. Phase lags are shown in red where the coherence squared is significant at 5% level.
